# Identifying risk factors for pediatric central-line–associated bloodstream infections

**DOI:** 10.1017/ash.2023.282

**Published:** 2023-09-29

**Authors:** Paula Conrad, Julie Murphy, Pascale Audain, Michelle Connors, Christopher Hopkinson, Jenny Chan Yuen, Jennifer Ormsby

## Abstract

**Background:** Pediatric patients often require central venous catheters (CVCs) for a variety of clinical indications, including medication administration, parenteral nutrition, and venous blood sampling. Patients with CVCs are at risk for central-line–associated bloodstream infections (CLABSI). These hospital-acquired infections are often preventable and may lead to increased morbidity and mortality. Clinicians at a 477-bed, freestanding pediatric academic hospital completed a quality improvement project to identify factors that place pediatric patients at increased risk for CLABSI and to outline strategies aimed at CLABSI reduction for our highest-risk patients. **Methods:** Project leaders completed a literature review to evaluate current research on the topic and then assembled a project team. The team completed a retrospective analysis and categorization of CLABSI cases and reviewed internal CLABSI root-cause analysis data. The group then completed a case–control analysis to identify risk factors in patients with CVCs who developed CLABSIs, compared to patients with CVCs who did not develop CLABSI. Following this analysis, the team created a CLABSI risk-factor tool for use by bedside nurses. This tool described patients with CLABSI risk factors and outlined best practices for CLABSI prevention. **Results:** Based upon literature review, root-cause analysis data, and retrospective CLABSI case review over the period from 2017 to 2021, an initial list of 9 potential CLABSI risk factors was compiled. A case–control analysis was performed comparing 97 CLABSI cases with 103 matched controls. Univariate, multivariate, and additional covariate analyses were employed to identify 3 factors placing pediatric patients at increased risk for CLABSI. These included (1) multiple enteral devices (ie, 2 or more devices, including gastrostomy tube, jejunostomy tube, gastrostomy or jejunostomy tube, ostomy, and peritoneal drain); (2) multiple CVC entries (ie, CVC used for medications and venous sampling); and (3) long-term CVC plus parenteral nutrition (CVC in place for >21 days and receiving parenteral nutrition and/or intralipids). **Conclusions:** Pediatric patients with central venous access are vulnerable to CLABSI, and certain patients may be at increased risk. Frontline clinicians may be able to identify these patients and adopt best practices to prevent infection. A tool for use by bedside nurses can be a useful adjunct to existing CLABSI prevention practices.

**Disclosures:** None

